# Whole genome sequencing facilitates intragenic variant interpretation following modifier screening in *C. elegans*

**DOI:** 10.1186/s12864-021-08142-8

**Published:** 2021-11-13

**Authors:** Francesca Jean, Susan Stasiuk, Tatiana Maroilley, Catherine Diao, Andrew Galbraith, Maja Tarailo-Graovac

**Affiliations:** 1 Departments of Biochemistry, Molecular Biology and Medical Genetics, Cumming School of Medicine, University of Calgary, Calgary, AB Canada; 2 Alberta Children’s Hospital Research Institute, University of Calgary, Calgary, AB Canada

**Keywords:** Mutagenesis screen, Intragenic modifier, Whole genome sequencing, *C. elegans*, CRISPR/Cas9

## Abstract

**Background:**

Intragenic modifiers (in-phase, second-site variants) are known to have dramatic effects on clinical outcomes, affecting disease attributes such as severity or age of onset. However, despite their clinical importance, the focus of many genetic screens in model systems is on the discovery of extragenic variants, with many labs still relying upon more traditional methods to identify modifiers. However, traditional methods such as PCR and Sanger sequencing can be time-intensive and do not permit a thorough understanding of the intragenic modifier effects in the context of non-isogenic genomic backgrounds.

**Results:**

Here, we apply high throughput approaches to identify and understand intragenic modifiers using *Caenorhabditis elegans*. Specifically, we applied whole genome sequencing (WGS) to a mutagen-induced forward genetic screen to identify intragenic suppressors of a temperature-sensitive *zyg-1(it25)* allele in *C. elegans*. ZYG-1 is a polo kinase that is important for centriole function and cell divisions, and mutations that truncate its human orthologue, PLK4, have been associated with microcephaly. Combining WGS and CRISPR/Cas9, we rapidly identify intragenic modifiers, show that these variants are distributed non-randomly throughout *zyg-1* and that genomic context plays an important role on phenotypic outcomes.

**Conclusions:**

Ultimately, our work shows that WGS facilitates high-throughput identification of intragenic modifiers in clinically relevant genes by reducing hands-on research time and overall costs and by allowing thorough understanding of the intragenic phenotypic effects in the context of different genetic backgrounds.

**Supplementary Information:**

The online version contains supplementary material available at 10.1186/s12864-021-08142-8.

## Background

Intragenic modifiers (in-phase, within-gene modifiers) can have drastic effects on the phenotypic output of a primary phenotype-causing variant. For instance, it has been recently discovered that a particular variant in *ABCA4,* which occurs at a high frequency in control populations, behaves pathogenically when in combination with an *in cis* secondary variant in *ABCA4* [1]. While understanding how these two variants interact together therefore informs clinical diagnoses, in most cases, it is challenging to disentangle the role of second-site variants, particularly in cases where statistical significance is challenging to reach, such as in rare disease patients. To tackle this, one method has been to study the enzymatic activity of the alleles in cell lines to get an appreciation of how the variants may interact [2]. However, this method is disadvantaged by the fact that cell lines are isogenic, meaning that even if a genetic interaction is found, it is unclear how this might be impacted by genomic context. A more robust method has been the use of unbiased mutagenesis modifier screens, which generate several hundred random point mutations throughout the genome. Depending on the primary phenotype-causing allele, many of these screens isolate intragenic modifiers, which can subsequently be used to shed some light onto how intragenic modifiers behave in different genetic contexts.

Unbiased mutagenesis modifier screens have been performed in *Caenorhabditis elegans* (*C. elegans)* for years to uncover novel genetic interactions or inputs into a biological pathway of interest. However, although intragenic modifying variants are frequently found, they are rarely the focus of such studies. Instead, the focus tends to be on the discovery of novel players in a pathway. Therefore, in a typical screen, it is standard to apply a mutagen, filter out strains containing intragenic variants by using Sanger sequencing, and then screen through the remaining strains, which presumably contain extragenic modifying variants of interest. The primary analysis goes into understanding how these extragenic variants, and the genes in which they are found, modify the primary phenotype-causing locus.

As expected then, technological advances in the field have focused on expediting the discovery of extragenic modifying variants. For instance, while traditional identification methods included mapping loci and targeted genomic sequencing, improved accessibility and affordability of whole genome sequencing (WGS) has led to its increased use in modifier screens to identify causal variants. However, while the use of WGS has been used successfully to identify extragenic modifying variants following either backcrossing or mapping strategies for many years now, the adoption of next generation sequencing technologies in mutagenesis screens has been slow, with many labs still relying on more traditional methods to find extragenic modifiers [3–6]. Considering this, it is perhaps unsurprising that many labs have been hesitant to adopt WGS approaches in the initial screening stages.

Here, we illustrate that the screening of intragenic modifiers benefits from using a WGS approach by performing a suppressor screen on a temperature-sensitive *zyg-1* allele and submitting for sequencing without any prior backcrossing to remove extraneous variants or outcrossing to mapping strains. In *C. elegans, zyg-1* encodes a polo kinase that is responsible for initiating centriole duplication and driving cell divisions [7]. Accordingly, temperature-sensitive mutations in *zyg-1* result in embryonic lethality at the restrictive temperature; strains containing suppressing variants can be easily screened for based on viability [8]. The human orthologue of *zyg-1* is *PLK4*; recessive truncating mutations in *PLK4* are known to cause microcephaly, primordial dwarfism, and chorioretinopathy, making the identification and understanding of modifiers of this gene clinically relevant [9]. By sequencing the strains generated in this screen and using in-house designed bioinformatics methods, we identify strains with intragenic suppressing variants and demonstrate that they account for approximately 12% of the total number of suppressing variants. We also emphasize that the identification of identical variants in intragenic suppressing strains is a measure of screen saturation. Finally, we show that having a complete picture of the genome of intragenic suppressing strains informs phenotypic output. Altogether, this work stresses the usefulness of WGS as a method for the discovery of intragenic modifiers in mutagenized strains.

## Results

### Overview of mutagenesis screen and isolation of suppressing strains

**Fig. 1:**
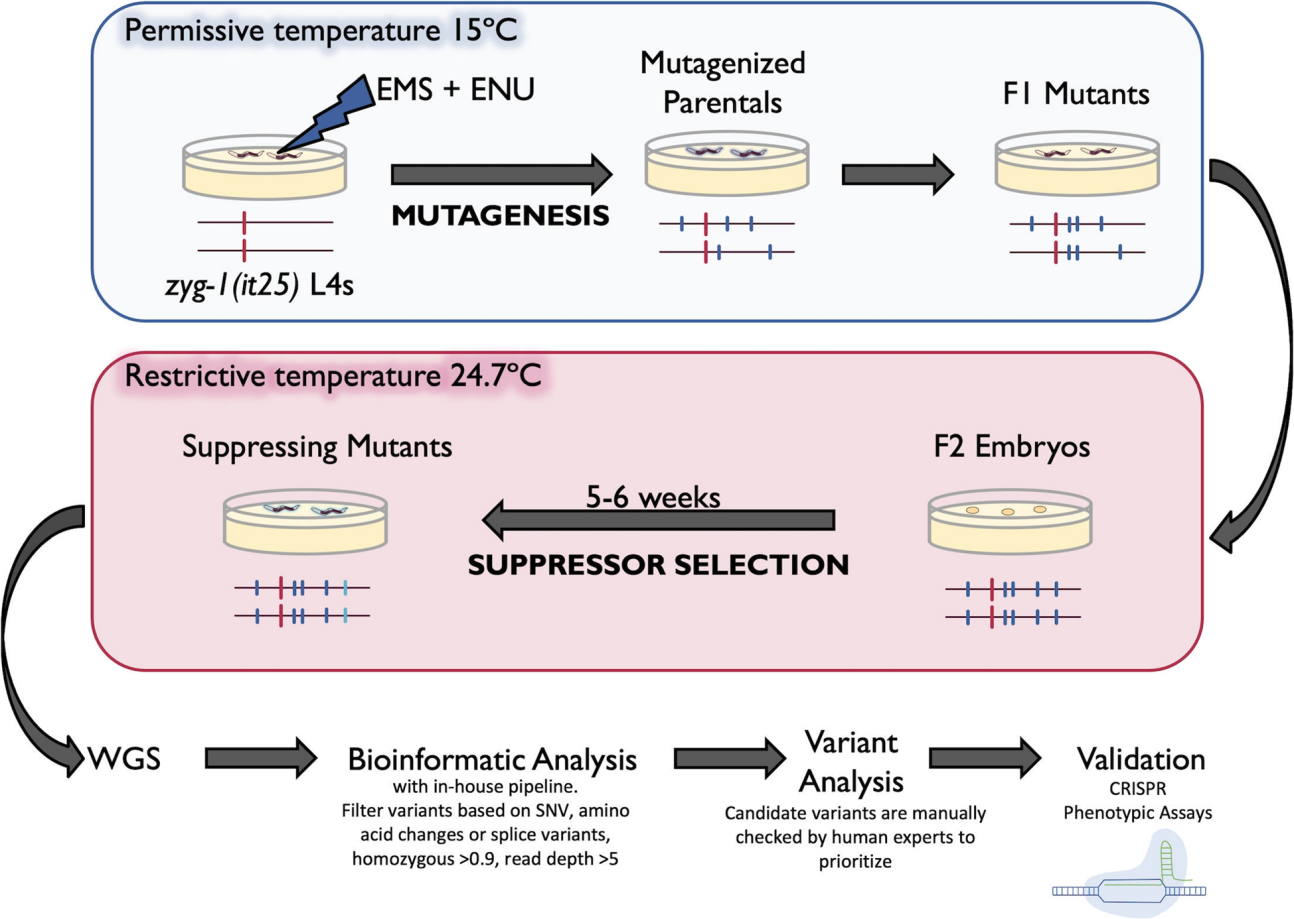
Overview of the mutagenesis screen. In brief, synchronized L4 *zyg-1(it25)* worms were exposed to EMS, ENU, or a cocktail of the two mutagens. Following incubation at the permissive temperature, F2 embryos were harvested and transferred to the restrictive temperature for 5–6 weeks to select for suppressing mutants. Homozygous populations that were validated to be suppressors had their gDNA extracted and were submitted for whole-genome sequencing (WGS). WGS files were analyzed using an in-house bioinformatics pipeline to produce a short-list of variants and these variants were manually curated to identify intragenic suppressing candidates. Finally, candidate intragenic variants were validated using CRISPR and phenotypic assays

*zyg-1(it25)* contains a missense mutation (P442L) in the Polo domain of ZYG-1, which is a master regulator of the centriole biogenesis pathway [8]. Accordingly, since pairs of centrioles form centrosomes which are an essential component of the mitotic spindle, mutations in *zyg-1* thereby inhibit cell division and lead to embryonic lethality [7, 10]. At the permissive temperature of 15 °C, *zyg-1(it25)* hermaphrodites lay viable eggs, but at the restrictive temperature of 24.7 °C, embryos have reduced viability (5.24% hatching rate) and are sterile, leading to no population survival (Table 2 and Fig. 3C). To isolate suppressors of *zyg-1(it25)*, we modified a robust and sensitive suppressor screen originally designed by Kemp et al [8]. First, synchronized L4 hermaphrodites were exposed to EMS and/or ENU (Fig. 1). EMS mainly generates GC to AT transition mutations whereas ENU predominantly generates A to T and T to A transversion mutations but also produces transition mutations; both were used to identify the widest variety of suppressing variants [11]. Homozygous F2 eggs were collected from gravid F1 animals and raised at the restrictive temperature for several generations to ensure the ongoing survival of worms harbouring suppressing variants and the death of populations lacking suppressing variants. Plates with surviving populations had one worm selected to propagate. Approximately 1.2 million haploid genomes were screened.

**Fig. 2:**
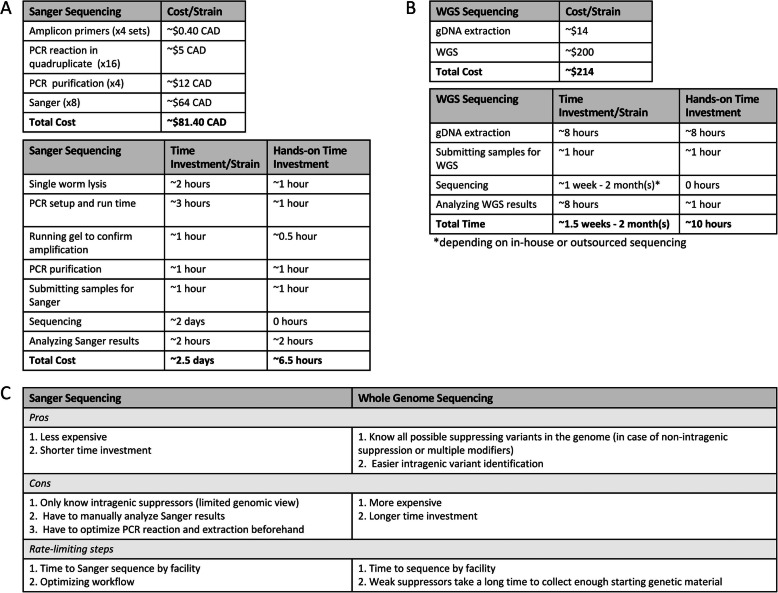
Comparison between Sanger and whole genome sequencing. A. Cost and time investment in using Sanger sequencing to identify strains containing intragenic suppressing variants. B. Cost and time investment in using whole genome sequencing to identify strains containing intragenic suppressing variants. C. Pros, cons, and rate limiting steps for Sanger and whole genome sequencing

Following mutagenesis screens, PCR amplification of intragenic regions and Sanger sequencing is typically used to identify intragenic variants. In the case of *zyg-1*, this requires four sets of amplicon primers to cover the coding region in order to test whether any intragenic variants are present and a substantial time investment in order to prepare each of the strains for Sanger sequencing. If we were to test a total of one hundred suppressor strains, the total cost per strain would work out to approximately $0.40 CAD for primers, $5.00 CAD for the PCR reaction, $12.00 CAD to purify the DNA, and $64 CAD to submit for Sanger sequencing (Fig. 2A). The total cost to test all one hundred suppressor strains would total about $8140 CAD and requires about a 2.5 day time investment (Fig. 2A). This is of course contingent upon gene size and sequence; for instance, large genes, or those with many exons, may require more primer sets and more PCR products must be purified and submitted for Sanger sequencing, which can dramatically increase costs. Similarly, sequences that are challenging to amplify may require substantial optimization. In our approach, however, we decided to forego the standard method and instead used WGS followed by the use of an in-house bioinformatics pipeline briefly described in the Materials and Methods to identify intragenic variants of interest. Specifically, all suppressing strains were sent for WGS, without prior backcrossing or outcrossing (Fig. 1). In our case, extracting genomic DNA costs approximately $14 CAD per strain and current rates for WGS (will vary depending on facility) average to ~$200 per strain; the total cost to submit each of the strains for WGS equals about $21,400 CAD (Fig. 2B), with the total hands-on time investment being about 7 h when the focus is on intragenic variants only (Fig. 2B).

After sequencing each of the suppressing strains generated through six separate rounds of mutagenesis, we found that a total of fifteen strains contained modifying variants in either the coding region of *zyg-1* or just upstream, representing approximately 12% of the total number of suppressing strains isolated in our screen. Therefore, although using PCR and Sanger to identify intragenic modifiers is seemingly cheaper than performing WGS, when we consider that intragenic modifiers only account for a fraction of the total number of suppressor strains in our screen, WGS is often still necessary for many strains to identify extragenic modifying variants or to explain differences between strains with identical intragenic modifying variants, as we see with several of our strains (Fig. 2C). Similarly, although performing PCR and preparing samples for Sanger sequencing can be performed in batches to expedite the process, samples must largely be analyzed individually (Fig. 2A). Conversely, not only can the genomic DNA extractions be performed in batches, but our pipeline also expedites the identification of intragenic modifiers, with users able to screen for variants quickly and easily in *zyg-1* using search functions (Fig. 2B). In fact, all strains can be screened for both the retention of the original *zyg-1(it25)* mutation and the presence of intragenic variants within minutes. Therefore, from both time and cost perspectives, WGS offers significant advantages over the more traditional methods of identifying intragenic modifiers (Fig. 2C).

### Spectrum of intragenic variants within zyg-1

Following the analysis of the output files, we determined that both mutagens were capable of generating intragenic suppressing variants, although we found that most of the strains containing intragenic variants were isolated in screens that included the addition of ENU. On average, the use of both EMS and ENU increased the number of filtered variants than either mutagen on its own, thereby increasing the total number of variants to screen through (Table 1). As would be expected by the mutational profile of the mutagens, many of the mutations were transition mutations, although we did find a few examples of transversion mutations (Table 1).

**Table 1:** Overview of strains containing intragenic variants

Strain	Mutagenesis	Filtered Variants	Position(s)	Ref/Alt	Variant Effects
*N2*	N/A	N/A	N/A	N/A	N/A
*zyg-1(it25)*	N/A	N/A	N/A	N/A	N/A
*MTG57*	EMS	122	5,651,146	C/T	c.1154C > T; p.A385V
*MTG192*	ENU	65	5,650,939 5,650,947	C/A T/G	c.947C > A; p.P316H c.955 T > G; p.S319A
*MTG308*	EMS/ENU	188	5,651,254	G/A	c.1262G > A; p.G421E
*MTG309*	EMS/ENU	176	5,651,146	C/T	c.1154C > T; p.A385V
*MTG315*	EMS/ENU	174	5,649,756	G/A	c.-18G > A
*MTG320*	ENU	73	5,651,219 5,651,252	T/A T/C	c.1227 T > C; p.H409= c.1260 T > A; p.N420K
*MTG329*	ENU	84	5,653,611	A/G	c.1597A > G; p.K533E
*MTG354*	ENU	78	5,650,847	A/C	c.938A > C; p.D313A
*MTG355*	ENU	70	5,651,254	G/A	c.1262G > A; p.G421E
*MTG381*	EMS/ENU	70	5,653,339	T/C	Revertant
*MTG398*	EMS/ENU	47	5,649,770	T/A	c.-4 T/A
*MTG406*	EMS/ENU	126	5,651,146	C/T	c.1154C > T; p.A385V
*MTG423*	EMS/ENU	123	5,651,073	G/A	c.1081G > A; p.E361K
*MTG426*	EMS/ENU	116	5,650,948	C/T	c.956C > T; p.S319F
*MTG437*	EMS/ENU	111	5,650,846	G/A	c. 937G > A; p.D313N

Of the fifteen strains containing putative intragenic suppressing variants, two strains contained two secondary mutations in *zyg-1*, twelve contained one secondary mutation, and one was a revertant of the original *zyg-1(it25)* mutation (Table 1). The majority of the candidate intragenic variants were nonsynonymous missense variants, however, there were two exceptions. One strain, MTG320, contained a synonymous *zyg-1* variant (H409=) that cooccurred with another candidate nonsynonymous variant (N420K) (Table 1). We predict that the nonsynonymous variant in this strain confers most, if not all, of the suppression effects. The second exception was that two strains, MTG315 and MTG398, contained single nucleotide changes in the 5′ untranslated region (UTR) of *zyg-1* (Table 1). These two variants occurred within 18 and 4 nucleotides of the start codon, respectively. A 5′ UTR variant was also uncovered in the Kemp et al. screen for suppressors of *zyg-1(it25)*, underscoring the importance of screening for suppressing variants within the UTRs of candidate genes, in addition to missense mutations within coding regions [8].

**Fig. 3:**
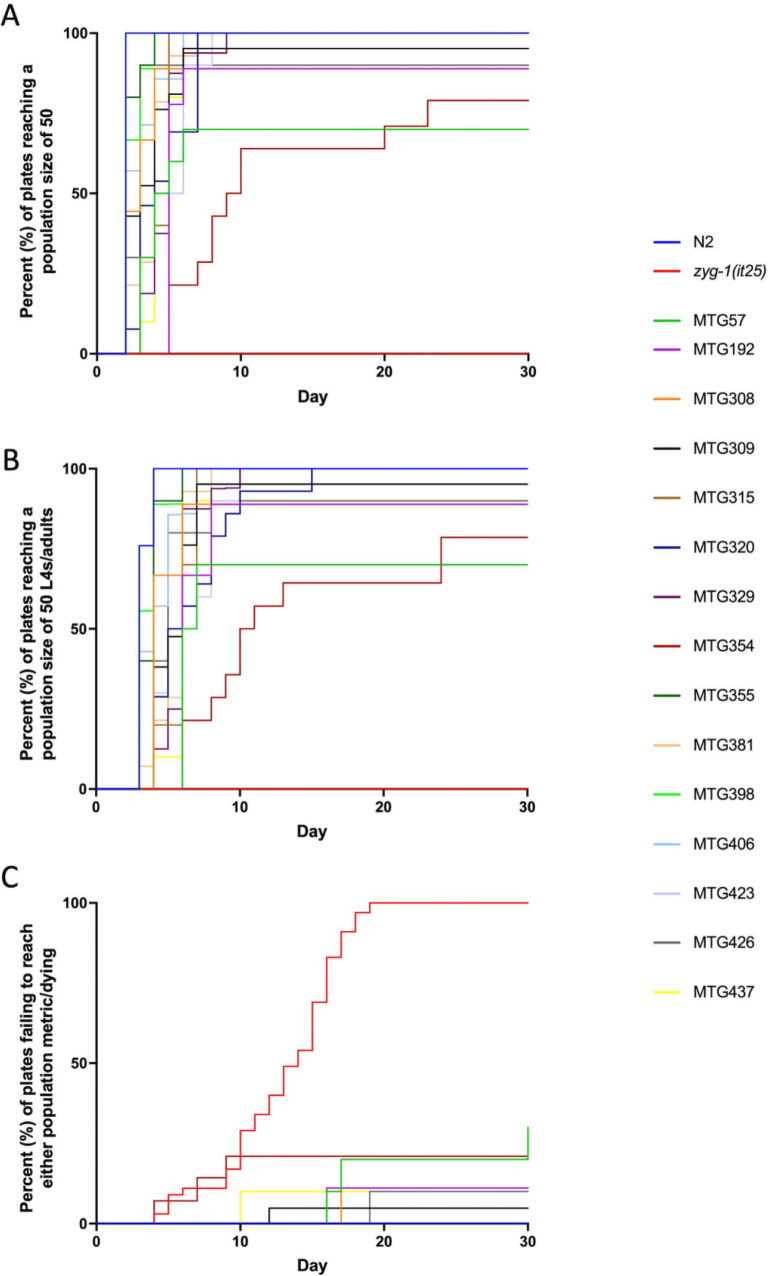
Summary of suppression strength of each of the strains containing intragenic secondary variants. A-C. Summary of population assay. Between 7 and 33 plates were tested for each genotype. A. Kaplan-Meier graph showing the percentage of plates of each genotype to reach a population size of 50. B. Kaplan-Meier graph illustrating the percentage of plates of each genotype to reach an L4 or adult population size of 50. C. Kaplan-Meier graph showing the percentage of plates of each genotype that ultimately resulted in population death. Plates that had not reached either of the population size metrics or had died out by 30 days were considered terminated. Both the hatching and population assays were performed at 24.7 °C unless otherwise specified

While each of the suppressor strains was selected based on their ability to survive at the restrictive temperature following a 6-week incubation period, we sought to quantify their suppression ability by using both hatching and population assays. At 24.7 °C, N2 has a high hatching rate, is able to lay around 200 progeny, and is able to generate a population of 50 worms within 2 days and a population of 50 L4 or older worms within 3 days (Table 2 and Fig. 3A-C). Conversely, *zyg-1(it25)* worms lay 50 worms less on average than N2 and only a small fraction of these are viable; accordingly, these worms are never capable of producing a population of 50 and eventually die out (Table 2 and Fig. 3A-C). The intragenic suppressor strains had varying levels of suppression at 24.7 °C, ranging from hatching rates only slightly higher than *zyg-1(it25)* on its own, such as 9.89% in MTG192, to surpassing wildtype N2 levels, such as 98.76% in MTG381 (Table 2). Furthermore, when analyzing population growth, we see that the suppressing strains are right-shifted in comparison to N2, which indicates that they do not reach the population metrics as quickly as the wildtype N2 strain (Fig. 3A-C). The weaker suppressor strains highlight one limitation to our approach; weak suppressors often grow poorly at the restrictive temperature, making it challenging to collect enough starting sample for WGS. Although we were able to collect sufficient starting samples for each of the suppressing strains, Sanger sequencing may be a more viable option for mutagenized strains that have low viability or strains may have to be grown at more permissive temperatures to generate enough material (Fig. 2C).

**Table 2:** Hatching rates for each of the strains containing intragenic variants

Strain	Average Eggs Laid	Hatching Rate	N
*N2*	188.68	89.48%	41
*N2 15°C*	262.4	97.29%	5
*zyg-1(it25)*	167.8	2.31%	49
*MTG57*	109	66.17%	15
*MTG192*	250.6	9.89%	10
*MTG308*	199.89	67.42%	9
*MTG309*	216.1	68.97%	16
*MTG315*	55.11	95.35%	9
*MTG320*	137.23	42.17%	13
*MTG329*	148.65	20.49%	17
*MTG354*	250.33	11.21%	12
*MTG355*	130.11	90.98%	9
*MTG381*	47	98.76%	9
*MTG398*	111.5	87.05%	10
*MTG406*	99	82.57%	10
*MTG423*	106.8	51.34%	10
*MTG426*	118.5	41.43%	10
*MTG437*	152.2	64.74%	10

### Analyzing the spectrum of intragenic variants may be used as a proxy for measuring screen saturation

In *C. elegans* mutagenesis screens in the past, screen saturation has typically been measured by determining whether the same gene or complementation group has been hit multiple times. Reaching saturation ensures that the maximum number of target genes have been identified. However, reaching saturation can be a cumbersome process, with traditional methods often requiring mapping modifying variants and performing complementation tests. This can prohibit the use of high-throughput modifier screens, with many labs instead screening through fewer haploid genomes to avoid being overwhelmed with strains. Here, we propose using the spectrum of intragenic variants to assess screen saturation, such as the occurrence of rare revertant events and of the same position being hit multiple times.

In our study, we were able to screen through over 1 million haploid genomes, a process that was made possible by performing whole genome sequencing on all suppressor strains and using the spectrum of intragenic variants as a proxy for saturation. One of the strains that we uncovered in our screen contained a putative reversion event of the original *zyg-1(it25)* allele (Table 1). Using traditional methods such as PCR and Sanger to identify this variant may have led to the potential classification of this strain as wildtype contamination. However, WGS provided evidence that this strain contained a true revertant since mutational events were observed. Specifically, WGS analysis revealed 70 coding variants within this strain and each of these variants matched the mutational profile of EMS and ENU, which would not have been able to be confirmed without using WGS. Furthermore, we saw several instances of the same position being repeatedly hit in our mutagenesis screen. For example, the same C > T mutation at position II:5651146 occurred three times in MTG57, MTG309, and MTG406, and the same G > A mutation at position II:5651254 occurred twice in MTG308 and MTG355. We also saw a couple examples of the same codon being hit, such as in MTG354 and MTG437, ultimately affecting the same protein position but resulting in different amino acid changes. Therefore, because we observed a rare reversion event and the same cDNA and protein positions being repeatedly hit within *zyg-1*, and the positions of our intragenic suppressing variants overlap with those found in the Kemp et al. (2007) screen in key functional regions, it is likely that our screen is nearing saturation [8].

### Intragenic variants are positioned non-randomly within zyg-1

**Fig. 4:**
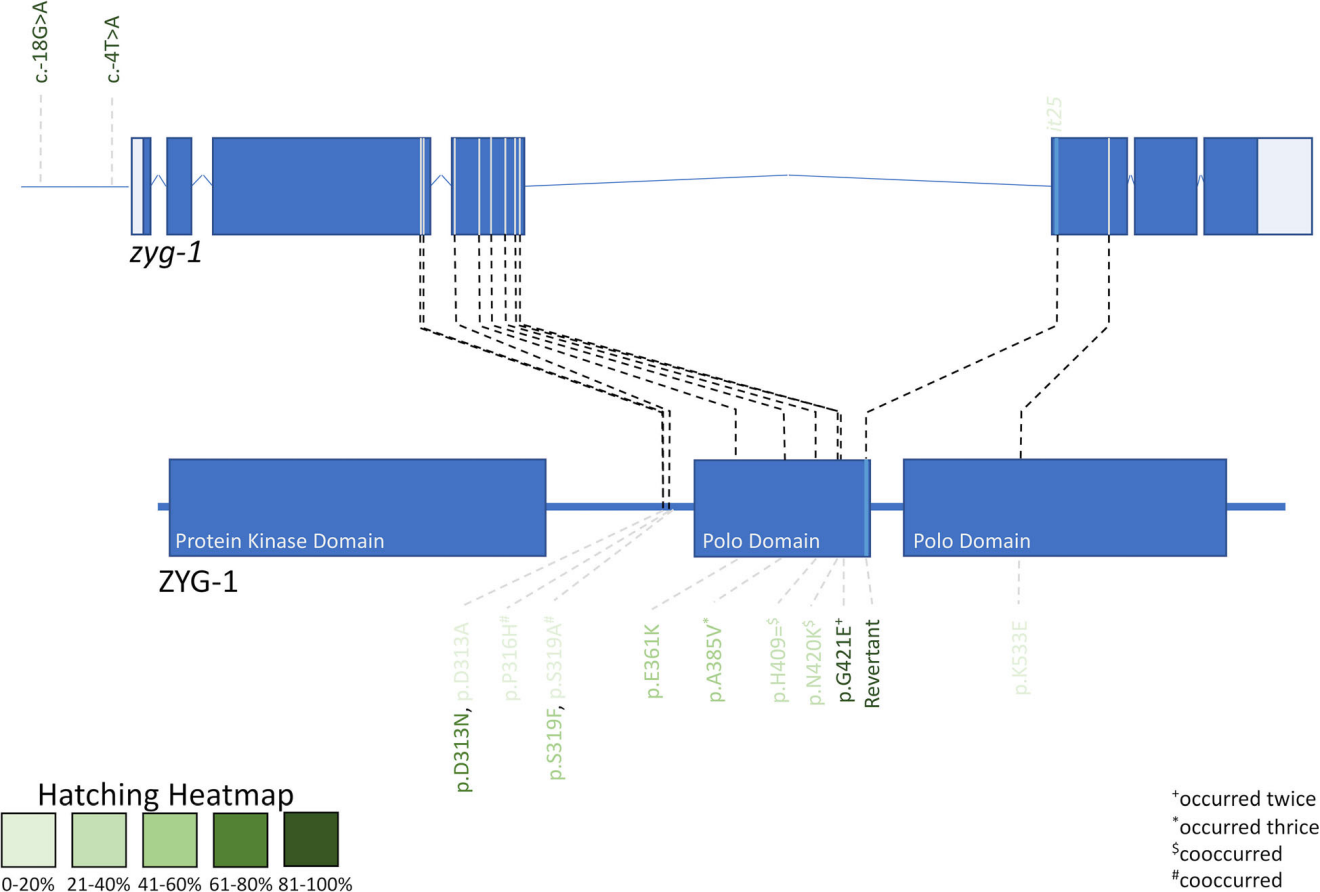
Summary of the positions of the intragenic variants within *zyg-1*/ZYG-1 and their relative hatching rates. The upper image shows the *zyg-1* gene arrangement along with the two 5′ UTR suppressing variants and the original *it25* variant. Below, the structure of the ZYG-1 protein is outlined, with the kinase and polo domains indicated in thick blue boxes and intragenic variants affecting the protein coding region listed underneath. Variants are colour-coded in green based on their hatching ability (confirmed through testing either the mutagenized strain or the CRISPR strain). Variants that occurred twice are indicated with a plus sign, variants that occurred thrice are indicated with an asterisk, and variants that co-occurred are indicated with either a dollar sign or hashtag

**Fig. 5:**
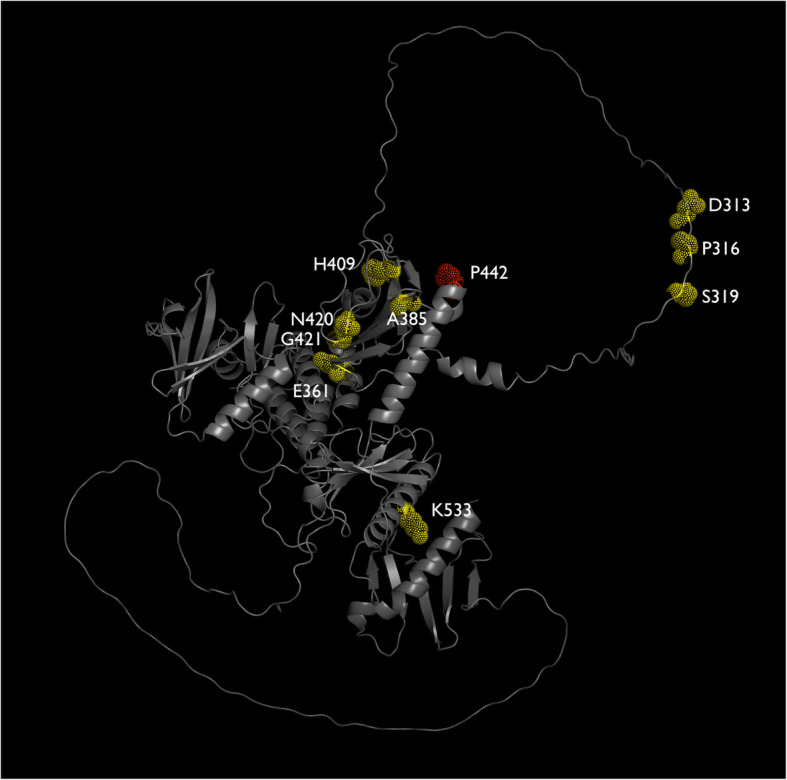
Predicted position of the intragenic variants within ZYG-1. Suppressing intragenic variants are coloured in yellow whereas the primary phenotype-causing *zyg-1(it25)* mutation is in red. Figure created with PyMOL with the predicted ZYG-1 structure downloaded from AlphaFold (ID: AF-Q9GT24-F1) [12, 13]

One current dilemma is interpreting and predicting the phenotypic effects of in-phase variants. Therefore, we next assayed whether the intragenic variants were distributed uniformly within *zyg-1*, as we predicted that the closer two variants are together, the more likely they encode directly interacting amino acids or amino acids that work together as part of a protein domain. The original *zyg-1(it25)* variant is a missense mutation (p.P442L) located within the polo domain (Figs. 4 and 5) [10]. As observed previously by Kemp et al., the secondary *zyg-1* variants that are capable of suppressing this variant are not uniformly distributed within the gene; the intragenic suppressing variants tend to cluster within, or just outside, the central polo domain or are present in the 5′ untranslated region (Figs. 4 and 5) [8]. We never uncovered any intragenic mutations located within the N-terminal protein kinase domain and only saw one example of an intragenic variant within the C-terminal polo domain. This reinforces the idea that two or more variants located within the same protein domain are more likely to have a genetic interaction and have a subsequent effect on protein function.

We next compared the specific position of the secondary variants within the *zyg-1* gene with suppressing ability, as it would be expected that certain variants would be able to suppress better than others. In general, the position of the variant within the *zyg-1* gene corresponded to the level of suppression, with variants located within the polo domain in proximity to the original *zyg-1(it25)* variant (such as A385V, H409=;N420K, G421E) tending to have higher rates of suppression, whereas variants located either further away in the polo domain (such as K533E) or outside of the polo domain (such as D313A and P316H;S319A) had moderate or low suppression rates. However, this association was not always clear-cut and often required further analysis. Indeed, we found three characteristics that must be taken into consideration when comparing the position of the variant to suppression ability: 1. strains containing the same intragenic variant do not always show the same suppression ability, 2. the specific amino acid change can have dramatic effects on suppression ability, and 3. variants in the UTR are essentially exceptions to the rule.

The first characteristic was that we encountered two cases where strains contained the same intragenic variant yet had vastly different hatching rates, meaning we could not instantaneously interpret the true suppression ability of that variant. For instance, MTG57, MTG309, and MTG406 contain the same A385V variant, yet have hatching rates of 66.17, 68.97, and 82.57%, respectively (Table 2). To tease apart the true contribution of this variant, we chose to use CRISPR and homology-directed repair to recreate this variant in the *zyg-1(it25)* background. In brief, guide RNAs close to the site of the variant were identified and then donor constructs were designed to include the candidate intragenic variant and synonymous changes to either the PAM or the guide RNA binding site. Following completion of the PCR and differential cutting genotyping method and confirmation with Sanger sequencing, homozygous strains were then tested for their ability to suppress the *zyg-1(it25)* mutation using both the hatching and population growth assays. As predicted since we observed this variant three times, recreating this variant in the *zyg-1(it25)* background was sufficient to suppress the original lethal phenotype, with worms having a hatching rate of 48.02% (Table 3), which is lower than the hatching rates of the mutagenized strains, suggesting that there may be additional variants in each of these strains that are able to boost their suppression ability. Another possibility is that the synonymous mutations present to disrupt rebinding of the guide RNA following insertion may be impacting function. To test for this possibility, the same guide RNA and donor construct were injected into the N2 background. The presence of the A385V mutation in the wildtype background did not appear to have a noticeable effect on viability. At 15 °C and 24.7 °C, these worms had a hatching rate of 93.13 and 94.78%, respectively, which is comparable to that of N2 (hatching rate of 97.29 and 89.48%, respectively) (Tables 2 and 3). Therefore, not only can we conclude that the A385V variant behaves as a silent genetic modifier, in that it has no detectable fitness effect when isolated from the primary modifier, but the true suppression ability of this variant is lower than originally predicted. Accordingly, we indicated the hatching rate of the CRISPR variant in the heatmap comparing the suppression ability to position of the variant within ZYG-1 (Fig. 4).

**Table 3:** Summary of CRISPR-generated strains verifying a subset of candidate intragenic and extragenic suppressing variants

Gene	Originating Strain	Injected Strain	Variant Effects	Hatching Rate (N)	Suppressor?	Phenotype	Strain Name
*zyg-1*	*MTG57*, *MTG309*, and *MTG406*	*zyg-1(it25)*	p.A385V	48.02% (5)	Yes	Viability	*MTG470*
*zyg-1* 24.7 °C	*MTG57*, *MTG309*, and *MTG406*	*N2*	p.A385V	94.78% (5)	N/A	Viability	*MTG538*
*zyg-1* 15 °C	*MTG57*, *MTG309*, and *MTG406*	*N2*	p.A385V	93.13% (5)	N/A	Viability	*MTG538*
*zyg-1*	*MTG320*	*zyg-1(it25)*	p.N420K and p.H409=	37.18% (8)	Yes	Viability	*MTG498*
*zyg-1* (from 15 °C)	*MTG329*	*zyg-1(it25)*	p.K533E	0.19% (8)	No	Embryonic Lethality	*MTG500*
*zyg-1* (from 20 °C)	*MTG329*	*zyg-1(it25)*	p.K533E	4.09% (5)	Yes	Viability	*MTG500*
*zyg-8*	*MTG329*	*zyg-1(it25)*	p.D31V	0% (4)	No	Embryonic Lethality	*MTG531*
*aak-2*	*MTG329*	*zyg-1(it25)*	p.G180R	0% (5)	No	Embryonic Lethality	*MTG541*
*him-19*	*MTG329*	*zyg-1(it25)*	p.P216S	1.09% (5)	No	Embryonic Lethality	*MTG537*
*zyg-1*	*MTG398*	*zyg-1(it25)*	c.-4 T/A	82.09% (7)	Yes	Viability	*MTG542*
*zyg-1/+*	*MTG398*	*zyg-1(it25)*	c.-4 T/A	19.48% (7)	Yes	Viability	*MTG542/zyg-1(it25)*

In another example, the G421E variant is carried in both MTG308 and MTG355, yet these two strains have a hatching rate that differs by over 20%; MTG308 had a hatching rate of 67.42% whereas MTG355 had a hatching rate of 90.98% (Table 2). Fortunately, this variant was also found in the Kemp et al. screen and was found to have a hatching rate of nearly 100%. Given that MTG355 and the strain found in the Kemp et al. screen had comparable hatching rates, we concluded that this variant typically has a high suppression ability and indicated so in the heatmap (Fig. 4).

A second characteristic was that the exact amino acid change can have dramatic effects on suppression ability. We isolated a couple examples of strains containing an intragenic variant that affected the same codon but resulted in a different amino acid change. For example, MTG354 and MTG437 both contain a missense variant at position D313, however the resulting amino acid is different (D313A and D313N, respectively) (Table 1). Despite the same amino acid position being affected in these two strains, they display vastly different suppression abilities. MTG354 is not a strong suppressor and only has a hatching rate of 11.21% and although 64% of individually plated worms will reach a population size of 50 within 10 days, 21% of worms will fail the assay, resulting in population death (Table 2 and Fig. 3A-C). In contrast, MTG437 has a hatching rate of 64.74% and subsequently is regularly capable of passing the population metrics, with only 10% of individually plated worms resulting in population death (Table 2 and Fig. 3A-C). Similarly, MTG192 and MTG426 are both affected at position S319; MTG192 has an alanine (S319A) at this position whereas MTG426 contains a phenylalanine (S319F) (Table 1). There is a striking phenotypic difference between these two strains though; MTG192 is the weakest intragenic suppressor found, with a hatching rate of only 9.89% and takes on average 5 days to reach a population size of 50 whereas MTG426 has a drastically higher hatching rate of 64.74% and reaches a population size of 50 within 2.67 days (Table 2 and Fig. 3A-C). Although it is obvious the difference in suppression ability between MTG192 and MTG426, it is important to consider that a direct comparison is challenging to make between these strains; MTG192 contains two *zyg-1* intragenic variants, meaning that it exists as a complex allele of P316H;S319A and may account for its reduced survival. Therefore, although we generally found that variants closer to the original *zyg-(it25)* mutation tended to have a higher suppression ability, this could be offset by the presence of particular amino acid changes.

A final consideration was that the 5′ UTR variants had profound suppression effects despite being the furthest from the original *zyg-1(it25)* mutation. To confirm that these upstream variants were indeed capable of suppressing the *zyg-1(it25)* mutation, we decided to recreate one of the variants and focused on MTG398, which contains a variant 4 nucleotides upstream of the start codon (c.-4 T/A). To re-create this allele, we decided against inducing either synonymous changes or changes to affect binding of the guide RNA and instead only created the upstream variant in the donor construct, since it was unknown whether any further changes would have an effect on viability. Upon recreating the variant in the *zyg-1(it25)* using CRISPR, we found that this strain had a hatching rate of 82.09% in the homozygous state, which is comparable to that of MTG398, which had a hatching rate of 87.05% (Tables 2 and 3). Based on this variant occurring upstream, we predicted that it would likely have an effect on the expression of *zyg-1*. Indeed, we found that the presence of this variant in the heterozygous state is also sufficient to suppress the original *zyg-1(it25)* mutation, albeit to a lower extent (hatching rate of 19.48%) (Table 3). Therefore, since this variant can suppress *zyg-1(it25)* as either a homozygote or heterozygote suggests that the upstream 5′ UTR variants may act by increasing the expression of *zyg-1*, which effectively compensates for the damaging *zyg-1(it25)* allele at the restrictive temperature. This contrasts with the missense alleles which we predict are more likely rescuing protein function.

### Genomic context, as provided by WGS, improves variant interpretation

EMS and ENU produce random point mutations in the genome, therefore, strains containing the same intragenic variant were not isogenic and accordingly, frequently had differences in suppression ability. This occurred between mutagenized strains containing the same variant and between the mutagenized strain and the recreated CRISPR strain. We predicted that these differences may be due to genomic context, therefore, we tested whether WGS could explain phenotypic differences in a subset of strains. In particular, we highlight in a few examples below that WGS allows us to detect genetic burden that may decrease viability and to probe whether candidate extragenic suppressors may enhance suppression dynamics.

As mentioned, MTG308 and MTG355 had different hatching rates at 24.7 °C despite containing the same intragenic variant (67.42 and 90.98%, respectively) (Table 2). Although we were able to compare these strains to a suppressor strain identified by Kemp et al. to discover the true suppression ability of this variant, WGS allowed us to assess the role that genomic context may play in the phenotypic differences between these two strains. For instance, after analyzing the genomes of each of the strains, it was found that MTG308 contained a total of 188 variants that satisfied our constraints whereas MTG355 contained only 70 variants (Table 1). The increased genomic burden in MTG308 by the presence of almost double the number of coding variants could be sufficient alone to explain its reduced viability, but we took a closer look at the types of variants present in the strains. In MTG308, we found that 5 of 188 variants were nonsense variants and 4 out of 188 affected splicing, representing 2.66 and 2.13% of the variants found, respectively (Supplemental Table 1). In contrast, MTG355 only had 1 nonsense variant out of 70 variants and 2 predicted splicing variants (representing 1.43 and 2.86% of the total variants, respectively) (Supplemental Table 1). Therefore, although the percentage of splicing variants is comparable between MTG308 and MTG355, MTG308 contains a greater proportion of nonsense variants that are likely causing a reduced fitness effect. With this explanation in mind, the G421E mutation is likely a strongly suppressing intragenic variant, which is supported experimentally since it was uncovered twice by us in our screen and was identified as a strongly suppressing variant in the Kemp et al. screen [8].

We next attempted to ask whether genomic context can explain phenotypic differences between the original mutagenized strain and the CRISPR-generated strain, with the prediction that we may be able to identify extragenic variants that are serving to enhance suppression dynamics. The first strain we tested, MTG320, was selected because it contained a complex allele (H409=;N420K) in which neither of the variants had been uncovered by either us or Kemp et al. [8]. Although the H409 = variant is largely predicted to not have a substantial suppression effect, the N420K missense mutation is adjacent to an amino acid change that has been found by both us and Kemp et al. [8]. As described, the G421E is able to strongly suppress the original *zyg-1(it25)* mutation so we decided to test whether the complex allele found in MTG320 was similarly able to strongly suppress *zyg-1(it25)*. After constructing the allele and subjecting it to our phenotypic assays, we find that this allele is in fact able to act as a suppressor and has a hatching rate of 37.18% (Table 3), which is comparable to the mutagenized strain MTG320 (hatching rate of 42.17%) (Table 2). Not only does this confirm that a variant at the N420 position is capable of suppressing the original *zyg-1(it25)* position, but it also suggests that this variant solely accounts for the suppression observed in MTG320 (ie. it is not aided by the presence of extragenic variants). Indeed, when screening through the list of 73 variants in this strain, we were unable to find any candidate extragenic suppressors, supporting the idea that the intragenic variant is the only suppressing variant in this strain.

In another example, there are phenotypic differences between the original mutagenized strain MTG329, which contains the only intragenic variant downstream of the *zyg-1(it25)* mutation, and the generated CRISPR strain. Following construction of the K533E variant in the *zyg-1(it25)* background, we found that this variant had a very low hatching rate of only 0.19% (Table 3) and was incapable of reaching any of the population metrics before the population becomes deceased (data not shown), suggesting that it is incapable of suppressing the *zyg-1(it25)* mutation on its own. In contrast, MTG329 had a hatching rate of 20.49% (Table 2), the K533E variant only contributes a fraction of this suppression ability, leading us to predict that there were other variants present that may enhance suppression in MTG329. To probe whether extragenic variants may account for the difference in hatching ability in the mutagenized strain MTG329 and the CRISPR strain containing the K533E variant, we manually screened through the WGS output files for candidate modifiers. We generated CRISPR strains individually containing plausible extragenic variants, including *zyg-8* (c.92A > T; p.D31V), which functions in spindle positioning, *aak-2* (c.538G > A; p.G180R), whose orthologue AMPK acts to regulate the expression of PLK4 in mouse embryonic fibroblasts, and *him-19* (c.646C > T; p.P216S), which functions in meiosis, but none of these variants were able to suppress the *zyg-1(it25)* allele on their own (Table 3) [14–16]. However, while we were unable to uncover any candidate extragenic variants that may enhance suppression dynamics, we noticed that this population seemed to survive at the restrictive temperature when we plated more than one worm at a time, suggesting that population survival may occur when worms become acclimated. We thus repeated both the population and hatching assays except instead of transferring the worms from 15 °C to the restrictive temperature, we grew the strain at 20 °C prior to testing at 24.7 °C. When completed in this manner, the hatching rate increased to 4.09% (Table 3) and we saw at least two individually plated worms reach a population size of 50 within a 30-day period (data not shown). Therefore, we conclude that the K533E variant is a weak suppressing variant that requires temperature acclimation to exert its full effects.

## Discussion

Here, we performed a chemical mutagenesis screen using a *zyg-1(it25)* allele in *C. elegans* combined with WGS and CRISPR/Cas9 to identify intragenic suppressors. We show that WGS facilitates the rapid identification of strains containing intragenic variants. Our approach expedites comprehensive interpretations of the phenotypic effects of each variant, and we show that having a complete view of the genome permits us to more accurately pinpoint the suppression contribution of each intragenic variant. For instance, the presence of substantial genomic burden can decrease a strain’s suppression ability. Moreover, by understanding the true suppression contribution of each variant, a clearer picture of the protein emerges.

The original *zyg-1(it25)* mutation causes a nonsynonymous change within the cryptic polo domain, which has been demonstrated to be necessary for SPD-2 binding and the subsequent recruitment of ZYG-1 to centrioles [17]. Specifically, the *zyg-1(it25)* mutation affects amino acid 442, where a proline is substituted for a leucine [8]. These amino acids have different biochemical properties, as proline is a cyclic molecule whereas leucine is hydrophobic, which likely disrupts the function of the cryptic polo domain. Indeed, disruptions in the cryptic polo domain have been shown to either completely abolish or substantially reduce centriole duplication, leading to the hypothesis that the *it25* allele reduces the ability of ZYG-1 to be recruited to the centriole to promote centriole biogenesis [17]. Considering that the vast majority of our intragenic suppressing variants also localize to the cryptic polo domain, it’s possible that these variants are able to restore its function and enhance the recruitment of ZYG-1 to the centriole. Indeed, our suppressor screen may provide a foundation to understand how both the *it25* mutation and the suppressing variants affect the conformation of ZYG-1 (Fig. 5) and how this impacts binding with key centriolar recruitment proteins; this approach has been successfully used previously to explain how conformational changes in AP2 impact binding with FCHo [18]. Similarly, considering that a heterozygous variant in the 5′ UTR is sufficient of rescuing the embryonic lethality phenotype, the upstream variants are conceivably increasing the expression of *zyg-1* thereby promoting the accumulation of ZYG-1 at centrioles.

Given that mutations in the human orthologue of *zyg-1*, *PLK4*, are associated with microcephaly and chorioretinopathy, this study highlights the importance of having a thorough understanding of the effects of combinations of variants within a given gene and determining whether variants occur in phase. Indeed, we find that secondary mutations that are closer to the primary phenotype-causing mutation are not only more likely to have a suppressive effect, but the effect is also more likely to be stronger. This is in line with work by Davis, Poon, and Whitlock (2009) which showed that intragenic compensatory mutations have a non-random distribution, with mutations that are closer to the primary mutation being more likely to be compensatory [19]. Although the effects of intragenic modifying variants have been largely understudied in disease dynamics, they may play a larger role than is currently recognized. For example, some studies have suggested that there may be up to nearly one hundred deleterious mutations in the genomes of otherwise healthy individuals, many of which are homozygous [20]. One possible explanation for the lack of disease in these individuals is the presence of compensatory mutations; in fact, Poon et al. discovered that for every deleterious mutation, they were able to find, on average, as many as 11.8 corresponding compensatory mutations, with the majority of these occurring as intragenic variants [21]. Indeed, upon testing one of the suppressing variants in both wildtype (N2) and sensitized backgrounds, we found that it showed no phenotype on its own but had substantial suppression ability when combined with the *zyg-1(it25)* allele, thereby highlighting the strong buffering, and typically non-toxic, attributes of these variants. Therefore, our work, in which we carefully studied the effects of intragenic suppressing variants in a clinically-relevant gene, has broad implications on interpreting complex alleles in disease.

Just as importantly, this study also emphasizes the importance of studying the effects of intragenic modifiers in the context of the genome. Although the use of cell lines or highly inbred laboratory strains can permit the study of complex alleles without having to consider genomic context, this is not representative of wildtype populations, which are typically non-isogenic. Our approach, in which we conducted an unbiased chemical mutagenesis screen to induce random point mutations throughout the genome, performed whole genome sequencing on suppressing strains, and then validated the ability of candidate intragenic variants to suppress the original phenotype-causing mutation is more representative of so-called “real-life” situations. Specifically, we were able to pinpoint the precise contribution of each intragenic variant in the context of diverse genomic backgrounds. This comprehensive analysis would not have been possible without the use of whole genome sequencing, emphasizing the usefulness of including NGS technologies in the analysis of intragenic modifiers.

Furthermore, we also highlight the many technical advantages to adopting WGS in intragenic variant identification. First, we show that overall, WGS is more cost and time effective than traditional Sanger sequencing approaches. With traditional methods, primers targeting the primary phenotyping loci must be designed and optimized. Then, each strain must have the locus amplified and Sanger sequenced, which is a very hands-on process, especially in cases where the primary gene is long or many modifying strains exist. Second, a major goal of modifier mutagenesis screens is to identify as many modifying targets as possible while simultaneously screening as few haploid genomes as possible, which can be measured by determining the degree of “saturation”. Using our approach, labs can use the spectrum of these variants to assess saturation (although this is limited to modifier screens using missense alleles as intragenic variants will not be uncovered in screens using deletion alleles). Third, since intragenic modifiers typically only account for a fraction of modifiers identified in a mutagenesis screen, WGS offers a rapid method to identify extragenic modifiers in the remaining strains.

### Conclusions

Here, we show that the analysis of intragenic suppressors generated from modifier screens benefits immensely from the incorporation of genome sequencing early in the process and CRISPR by subjecting strains generated from an unbiased chemical mutagenesis screen using a temperature-sensitive *zyg-1(it25)* allele to WGS, filtering for intragenic variants using an in-house pipeline, and validating with CRISPR and homology-directed repair. Specifically, we show that not only can intragenic variant discovery be expedited when using whole genome sequencing on suppressing strains, but our ability to interpret the suppression effects of each intragenic variant is enhanced. For instance, because modifier screens can more easily be completed to saturation, we can specifically determine which amino acids are more likely to genetically interact with the primary affected amino acid and to what extent they modify it. Additionally, by having a complete picture of the genomic context, we can accurately ascribe the contribution of each intragenic variant to a given strains ability to suppress the original mutation. Ultimately, this work serves to improve our ability to interpret gene-disease relationships by providing a methodology for the high-throughput identification of intragenic modifiers.

## Methods

### Mutagenesis screen

Six rounds of L4 *zyg-1(it25)* worms were exposed to 40 mM of ethyl methanesulfonate (EMS) and/or 1 mM *N*-ethyl-*N*-nitrosourea (ENU) for 4 h at room temperature on a bench-top rocker. Worms were subsequently washed, pelleted, and approximately 10 worms were picked onto ~ 100 plates; every plate was processed individually going forward. Eggs were isolated from gravid F1 worms using a sodium hypochlorite/sodium hydroxide solution. Isolated F2 eggs were split between 2 plates and incubated at 16 °C overnight to hatch. Plates were then shifted to the restrictive temperature of 24.7 °C with biweekly feeding of 10% w/vol OP50 in M9 buffer. After 6 weeks, plates were screened for surviving worms; one worm per plate was used to establish a suppressing population.

### Hatching rates and suppressing assays

For all phenotypic assays, L4 worms were individually plated and kept at the restrictive temperature of 24.7 °C unless otherwise noted. For the hatching assay, the worm was transferred the next day to a fresh plate and all progeny (hatched worms and unhatched, fertilized eggs) were counted. Following another 24 h, all hatched embryos were counted. This process was repeated for 4 days. Progeny counted from worms that died, crawled off the plate, or burrowed into the agar were included in calculating the total hatch rate. The ratio of the total number of hatched embryos to the total number of eggs laid was then calculated. For the population assay, viable worms on each plate were counted every day until the following population metrics were met and the resulting day at which the metric occurred was recorded: days until population of viable worms reaches 50 and days until population of L4 or adult worms equals 50, or until the worm population completely died. A population was considered dead when no worms were able to move spontaneously or respond to a touch stimulus. If plates had not reached any of the population metrics by 30 days, the experiment was terminated as the rate of worms dying typically equalled the rate of worms hatching by that time period.

### Genomic extraction

Genomic DNA from suppressing strains was extracted using the Qiagen Blood and Tissue kit (Cat #: 13323) following standard procedure [22]. DNA was suspended in 10 mM Tris-HCl (pH 8.0), only samples that had a minimum A260/280 ratio of 1.8 were submitted for sequencing. Genomic DNA was sent to the University of Calgary sequencing facility (Illumina NovaSeq 6000 or Illumina NextSeq 500) [https://www.ucalgary.ca/dnalab/], Genome Quebec (Illumina HiSeq PE150 or Illumina NovaSeq 6000 PE150) [http://gqinnovationcenter.com], or Sick Kids (Illumina NovaSeq 6000) [http://tcag.ca/facilities/dnaSequencingSynthesis.html].

### WGS analysis

WGS datasets were analyzed with a custom pipeline. Read sequences were trimmed with Trimmomatic v0.39 and trimmed fastq sequences were aligned with BWA-MEM v0.7.17 using the Wormbase *C. elegans* genome version WS265 as the reference [22–24]. Variants were called with Varscan v2.3.9 called variants and annotated by CooVar [25, 26]. Using a customized Perl script, the variants were filtered based on read depth (> 5), position within gene or proximity to splice site (coding region or +/− 10 nucleotides from a splice site), allele frequency (> 0.9 to select for homozygous genes), and de novo in comparison to the parental strain. Output files for each strain were manually screened through to ensure that each strain maintained the original *zyg-1(it25)* mutation and to identify any secondary intragenic variants.

### CRISPR injections

Guide RNAs were selected based on proximity and predicted efficiency from the UCSC genome browser [genome.ucsc.edu] and ordered as an Alt-R CRISPR-Cas9 gRNA from IDT (Table 4). Donor constructs (ssODNs) were designed using Horizon Discoveries tool [https://horizondiscovery.com/en/products/tools/Edit-R-HDR-Donor-Designer-oligo] and ordered as a 4 nM Ultramer DNA Oligo from IDT (Table 5). Primers flanking the site of insertion were ordered from IDT and used with a restriction enzyme based genotyping strategy and Sanger sequencing to confirm insertion (Table 6). A mix of 0.25 μg/uL of Cas9 (IDT; cat#: 1081058), 2.5 uM each of preannealed gRNA and tracrRNA, 5uM of the ssODN, and 40 ng/ul of an injection marker (*PRF4::rol-6(su1006)*) or a mix that had been diluted in half was injected into the gonads of *zyg-1(it25)* young adults. F1 adults positive for the injection marker were screened for heterozygous insertions using the designated genotyping method and F2 progeny from F1 injection-positive adults were tested for homozygous transmission to establish a population.

**Table 4:** Guide RNAs designed for *zyg-1* intragenic suppressors

Strain	gRNA	PAM
MTG309	GGTTTGAAGTTGCAGCTCAA	GGG
MTG320	AGTTGATGAAATGGTTCAAA	CGG
MTG329	CGAGCAATGTCTTAACGGAA	TGG
MTG398	TTGATCAACTATGAGATGAG	CGG

**Table 5:** Donor constructs (ssODNS) designed for re-creating intragenic suppressors using homology-directed repair

Strain	ssODN Sequence (5′➔3′)
MTG309	TATATTGTTGAATTGGATACTCGTTGTCGGTTTGAGGTAGTAGCTCAAGGGAATTTCGTTAAACGAATTTTGATTG
MTG320	GGATTGTTCGTCAACGTAATCAATTCTTCTTCTCCTTTTCTTCCGCGTACTGTTCTATCAGGTATTCGGTGTACATAAACTGTTTGAACCATTTCATCAACTTCGACAATCAAAAT
MTG329	CTGGAATAACACTTACAAAAGTGAATGAAGTATATGAATATCTAATAAGATTTGAACAATGTCTTAACGGAATGGATCGAGGAATGGTGTG
MTG398	ATCTTATTTTTAGCGCACCAAGTGTTGATCAACTAAGAGATGAGCGGTGGGAAGAGTGGTTCAAGATTGAG

**Table 6:** Genotyping strategy to confirm insertion of the donor constructs

Strain	Forward (5′➔3′)	Reverse (5′➔3′)	Length	Annealing T_**m**_	RE
MTG309	ACGACAGAGATCGAGGGAA	ACCGGAGGAGGATGTGAA	777 bp	61 °C	BbvI
MTG320	ACGACAGAGATCGAGGGAA	ACCGGAGGAGGATGTGAA	777 bp	61 °C	Hpy166II
MTG329	AGATGGTTGCTGTGACGATAAG	GGTACTCGATCAGTTCGCATAAA	792 bp	61 °C	BsrDI
MTG398	CCACTCTTTGTCCCACTCTAAA	CTCTTAATCGCCACCTTCTCTC	575 bp	52.5 °C	DdeI

## Supplementary Information

**Additional file 1.**

## Data Availability

The datasets generated and/or analysed during the current study are available in the NCBI BioProject repository (https://www.ncbi.nlm.nih.gov/bioproject/), PRJNA761686.
