# Supplementary material for: Whole genome sequencing facilitates intragenic variant interpretation following modifier screening in C. elegans
Source: BMC Genomics. 2021 Nov 13;22:820. doi: 10.1186/s12864-021-08142-8 (PMC8590768; doi:10.1186/s12864-021-08142-8)
Supplement: Supplementary file 1 — Additional file 1. [file 12864_2021_8142_MOESM1_ESM.docx]

***Supplementary Material***

**S1 Table. Comparison of the number of nonsense and splicing variants between MTG308 and MTG355.**

|  | **MTG308** | | **MTG355** | |
| --- | --- | --- | --- | --- |
|  | **Found** | **Ratio** | **Number** | **Ratio** |
| Nonsense | 5/188 | 0.02659574 | 1/70 | 0.01428571 |
| Splicing | 4/188 | 0.0212766 | 2/70 | 0.02857143 |
